# Acknowledgement to Reviewers of *Behavioral Sciences* in 2018

**DOI:** 10.3390/bs9010008

**Published:** 2019-01-11

**Authors:** Behavioral Sciences Editorial Office

**Affiliations:** MDPI, St. Alban-Anlage 66, 4052 Basel, Switzerland

Rigorous peer-review is the corner-stone of high-quality academic publishing. The editorial team greatly appreciates the reviewers who contributed their knowledge and expertise to the journal’s editorial process over the past 12 months. In 2018, a total of 115 papers were published in the journal, with a median time to first decision of 18 days and a median time to publication of 50 days. The editors would like to express their sincere gratitude to the following reviewers for their cooperation and dedication in 2018:
Agnew-Blais, JessicaGeorgian, BadicuOrlowski, DariuszAhmed, ZehraGericke, Niklas MarkusOverholser, JamesAllen, CoreyGoodill, SherryOzella, LauraAlleva, EnricoGoodman, SamanthaOzturk, YagmurAnderson, Nathaniel E.Grivokostopoulou, FoteiniPace, NelsonAndrade, Jeanette M.Gross, IsraelPage, Amy DellingerAngelou, AnastasiosGuina, JeffreyPanno, AngeloAnsa, BenjaminGulwadi, Gowri BetrabetPatel, PriyankaAsberg, KiaHagenauer, GerdaPathak, DhrubaAsnaani, AnuHandelman, MikaPauschenwein, JuttaAssari, ShervinHanstock, TanyaPazzaglia, FrancescaAtkins, RahshidaHarley, DavidPena-Shaff, JudithAttema, Arthur E.Hazell, Alan S.Pereira, LianeAtti, Anna RitaHeesch, KristiannPérez-Fuentes, María Del CarmenAttuquayefio, TukiHein, Sascha D.Peterson, DanielBachrach, AsafHicks, AnnPetrescu, MariaBadau, AdelaHo, Allen L.Phillips, AndelkaBadau, DanaHo, RogerPitteri, MarcoBaddam, Suman K. R.Hock, EmmaPluut, HelenBaeza-Velasco, CarolinaHofmeyr, AndrePolanska, KingaBailey, KylieHogge, Ingrid T.Polimanti, RenatoBalague Serre, NataliaHoran, KristinPoole, JayBalzan, RyanHovmand, Peter S.Posada-Quintero, HugoBanerjee, KalpitaHoward Wilsher, StephaniePoulton, Alison SallyBanerjee, SudipHuang, TonyPravst, IgorBarnes-Josiah, DeboraIgasaki, TomohikoPrice, JayneBast, NicoIimura, ShuheiPrieto-Lloret, JesusBazzigaluppi, PaoloInta, DragosPrior, SarahBeil, KurtInvitto, SaraPuertas Molero, PilarBekmuratova, SarbinazIsokpehi, RaphaelRaber, JacobBendick, MarcJackson, SarahRamirez, MarizenBenneyworth, MichaelJang, YunRauthmann, JohnBergbom, IngegerdJantzen, McNeel G.Regolin, LuciaBiasutti, MicheleJiang, ShanRennison, Callie MarieBilancini, EnnioJohnson, Lance A.Reybrouck, MarkBishop, AlexJones-Eversley, SharonRhee, Seung-YoonBlaser, ErikJoo, YuyoungRiar, Amanjot KaurBoardman, JedKalsi, NavkiranRittenour, Christine E.Bornstein, RobertKampourakis, KostasRivera, VictorBoucher, JulieKano, TakeshiRoberson, Donald N.Bowen, DeborahKaplan, Robert C.Robitaille, EricBrothers, BrittanyKapoulas, VaggelisRodriguez, EstrellaBrown, RhondaKaye, LindaRogobete, Alexandru FlorinBrowning, MatthewKey, AndreRogozea, LilianaBuksnyte-Marmiene, LoretaKeynan, IritRomero Martínez, ÁngelBunik, VictoriaKim, Hyun AhRuiz-Barquín, RobertoCapasso, RaffaeleKing, JulietRyabov, IgorCarney, AmyKirkland, KevinRydstedt, LeifCatherine, HayesKnight, HelenSá, Maria JoséCattaneo, AnnamariaKnouse, Laura E.Sætrevik, BjørnCerejeira, JoaquimKo, CelineSalari, SoniaChalah, MoussaKoch, Sabine C.Samadi, Sayyed AliChauhan, MunishKotloski, RobertSamaritter, RosemarieChauhan, SmitKoutná, VeronikaSandy, LarissaCheadle, AlyssaKristman, Vicki L.Sasangohar, FarzanCheng, JiaKrysinska, KarolinaSaurina, CarmeChhatbar, PratikKukula-Koch, WirginiaSavelyev, AlexanderChmielewski, SzymonKumar, ArvindSawyer, KeithChoi, KelvinKurti, Allison N.Scandurra, CristianoCohen, Harold C.Kwan, Mun YeeScharp, Kristina M.Coman, EmilKwan, SanSanSchermer, Julie AitkenConnor, Helene DianaLabrou, NikolaosSchuck, Sabrina Elayne BrierleyCossins, AnneLacey, SimonSerafini, GianlucaCothran, Dee LisaLafave, Lynne M.Z.Sergaki, MaritinaCounselman-Carpenter, ElisabethLane, Scott D.Servidio, RoccoCox, CodyLange, EwaShambley-Ebron, DonnaCrivelli, DavideLanglais, MickeySherman, Ryne A.Crook, Amy E.Lee, Byung CheolShintel, HadasCuberos, Ramón ChacónLee, Ya-WenShmargad, YotamCui, XiaoyingLeong, Wei ShinSiegel, Jessica A.Curry, GwenettaLesiuk, TeresaSiniscalco, DarioCutrona, CarolynLevy, FlorenceSmith, BrandtDagda, Ruben K.Li, ManyuSmith, Julie A.Dal Ben, MatteoLilenfeld, LisaSobrino, Xosé Antón VilaDale, RachelLiu, Wei-XiangSommerauer, Michael BernhardDavison, KarenLiu, YanSpagnoli, PaolaDelavega, ElenaLoi, NatashaStarościak, WojciechDeligeoroglou, EfthimiosLópez-López, DanielStary, ChristianDening, TomLoriol, MarcSticca, Fabioder Nederlanden, Christina M. Vanden BoschLuca, MariaŠtrac, Dubravka ŠvobDey, SoumyadeepLuck, EdwinaStrelnikov, KuzmaDezetter, AnneLynch, GeorginaSun, JiahongDharker, NachiketMacDonald, DouglasSuñer Soler, Maria RosaDi Nauta, PrimianoMahatmya, DuhitaSutton, JillDiaz, AnjoliiMaisha, BuumaTaverna, LiviaDiaz, MarvinMakecha, RadhikaTheorell, ToresDingemanse, MarkMakena, MonishTitlow, JoshDoddapattar, PrakashManzo, CiroTomasetti, CarmineDresp-Langley, BirgittaMartín-Hernández, DavidTopa, GabrielaDrobac, Jennifer A.Marzoli, DanieleTorquati, LucianaDuckham, BryanMathis, Mary W.Torregrosa Díez, María SoledadDundes, LaurenMcElrath, KarenTwedt, ElyssaDunphy, KimMcRae, Katerivan Amsterdam, Jan G.C.Ebstein, Richard P.Meinel, Christophvan den Noort, MauritsEhlen, J. ChristopherMeloni, BrunoVeeraragavan, SurabiEhrenberg, ShantelMich, OrnellaVelija, PhilippaEl Ghoch, MarwanMiller, LuisVenette, Steven J.Elkis-Abuhoff, Deborah L.Minazzi, RobertaVenta, Amanda CristinaElliott, LewisMitrofanis, JohnVersnel, HuibEllis, DavidMóczár, CsabaWagner, Norbert L.Enoiu, RazvanMolina-Mula, JesúsWard, Kenneth D.Evans, PatriciaMoores, CarlyWechsler, TheresaFacal, DavidMortiboys, HeatherWeinstein, Aviv M.Falchi, MarioNam, YoonjaeWeismayer, ChristianFenton, RachelNegri, NanetteWenos, JeanneFortgang, RebeccaNemec Zlatolas, LiliWestbury, Chris F.Francis, KarenNewins, AmieWong, Chi-kin KenchiFrazier, PatriciaNguyen, AnnWu, YouyouGaleazzi, Gian MariaNorcott, CandiceYang, XiaoGalfalvy, HangaNormann, Sven A.Yoon, JunghyunGander, PhillipO’Higgins, JamesYuan, BoGao, QiOkbay, AysuZarini, GustavoGarcia, OscarOlivas, Raul NavarroZhu, LinGarza, KimberlyOosthuizen, Jacques


